# Screening the Influence of Biomarkers for Metabolic Syndrome in Occupational Population Based on the Lasso Algorithm

**DOI:** 10.3389/fpubh.2021.743731

**Published:** 2021-10-12

**Authors:** Qiao-Ying Xie, Ming-Wei Wang, Zu-Ying Hu, Cheng-Jian Cao, Cong Wang, Jing-Yu Kang, Xin-Yan Fu, Xing-Wei Zhang, Yan-Ming Chu, Zhan-Hui Feng, Yong-Ran Cheng

**Affiliations:** ^1^Occupational Disease Department, Hangzhou Occupational Disease Prevention and Control Hospital, Hangzhou, China; ^2^Metabolic Disease Center, Affiliated Hospital of Hangzhou Normal University, Hangzhou, China; ^3^School of Mathematics and Statistics, Jiangsu Normal University, Xuzhou, China; ^4^Zhejiang Geriatric Care Hospital, Hangzhou, China; ^5^Neurological Department, Affiliated Hospital of Guizhou Medical University, Guiyang, China; ^6^School of Public Health, Hangzhou Medical College, Hangzhou, China

**Keywords:** lasso regression algorithm, metabolic syndrome, occupational population, biomarkers, physical examination

## Abstract

**Aim:** Metabolic syndrome (MS) screening is essential for the early detection of the occupational population. This study aimed to screen out biomarkers related to MS and establish a risk assessment and prediction model for the routine physical examination of an occupational population.

**Methods:** The least absolute shrinkage and selection operator (Lasso) regression algorithm of machine learning was used to screen biomarkers related to MS. Then, the accuracy of the logistic regression model was further verified based on the Lasso regression algorithm. The areas under the receiving operating characteristic curves were used to evaluate the selection accuracy of biomarkers in identifying MS subjects with risk. The screened biomarkers were used to establish a logistic regression model and calculate the odds ratio (OR) of the corresponding biomarkers. A nomogram risk prediction model was established based on the selected biomarkers, and the consistency index (C-index) and calibration curve were derived.

**Results:** A total of 2,844 occupational workers were included, and 10 biomarkers related to MS were screened. The number of non-MS cases was 2,189 and that of MS was 655. The area under the curve (AUC) value for non-Lasso and Lasso logistic regression was 0.652 and 0.907, respectively. The established risk assessment model revealed that the main risk biomarkers were absolute basophil count (OR: 3.38, CI:1.05–6.85), platelet packed volume (OR: 2.63, CI:2.31–3.79), leukocyte count (OR: 2.01, CI:1.79–2.19), red blood cell count (OR: 1.99, CI:1.80–2.71), and alanine aminotransferase level (OR: 1.53, CI:1.12–1.98). Furthermore, favorable results with C-indexes (0.840) and calibration curves closer to ideal curves indicated the accurate predictive ability of this nomogram.

**Conclusions:** The risk assessment model based on the Lasso logistic regression algorithm helped identify MS with high accuracy in physically examining an occupational population.

## Introduction

Metabolic syndrome (MS) refers to a group of metabolism-related diseases, including obesity, dyslipidemia, diabetes/impaired glucose tolerance, hypertension, and other diseases (1). The number of patients with MS has increased with the increasing number of obese patients worldwide (2). At present, the global prevalence of MS is about 25%, indicating that nearly one billion people are affected. Among these, the occupational population occupies a significant part and continues to increase (3). It has posed a substantial economic burden and has become a serious public health problem.

China ranks first in the world, with nearly 900 million working people. Every year, nearly 25 million workers suffer from health hazards, among which MS is already an important risk factor seriously affecting the health of the occupational population (4). Many studies were conducted on the relationship between the working environment of the occupational population and MS. Ma et al. confirmed that exposure to heavy metal elements in the work environment affected the body's metabolic function and increased the risk of MS in the Chinese population (5). (6) confirmed that the long-term exposure to noise in the work environment increased the chance of suffering from MS in the Chinese professional population (6). At the same time, some related studies confirmed the relationship of MS with the type of work in different occupational groups (7–9). Therefore, performing early MS screening for the occupational population is of great significance.

Machine learning, whereby a computer algorithm learns from prior experience, was recently shown to perform better than traditional statistical modeling approaches (10, 11). Machine learning algorithms have been widely used to screen biomarkers for related diseases with the rapid development of artificial intelligence (12–14). Various supervised machine learning models based on the least absolute shrinkage and selection operator (Lasso) regression algorithm have been successfully applied to medical data (15). However, no relevant studies used the Lasso algorithm to screen relevant biomarkers for MS.

Therefore, the risk of MS can be better predicted if the biomarkers related to MS are screened, and a risk prediction model is established for biomarkers used in routine physical examination. In this study, the Lasso logistic regression feature selection algorithm of machine learning was used to screen the biomarkers related to MS, and a risk prediction model was established.

## Materials and Methods

### Population and Data Collection

This study included occupational workers with operations in Zhejiang Province, China, between September 2010 and September 2020. The ethics committee of the Affiliated Hospital of Hangzhou Normal University approved all the procedures performed. The working environment included the metallurgical industry (35%), including steelmaking, ironmaking, steel rolling, coking, and so forth; casting, forging, heat treatment, and so forth in the machinery manufacturing industry (40%); and kiln workers and furnace workers in the glass and refractory industries (25%). A total of 3,077 workers were examined, of which 233 workers were excluded due to incomplete records and errors. Finally, 2,844 workers were selected for the study. According to relevant studies, related inflammatory factors, factors of erythrocyte parameters, blood pressure factors, lipid metabolic factors, obesity factors, and glucose metabolic factors are related to metabolic syndrome (16). This study included 32 basic biomarkers for routine physical examination in the population (Table 1). All the included people were physically examined by professional doctors according to the diagnostic criteria of MS (17) in the Chinese population.

**Table 1:** Types of medical markers included in the study.

**ID**	**Indicator name**	**ID**	**Indicator name**	**ID**	**Indicator name**	**ID**	**Indicator name**
1	Red blood cell count	9	Large platelet ratio	17	Percentage of monocytes	25	Total bilirubin level
2	Total protein level	10	Red blood cell distribution width correlation variance (CV)	18	Leukocyte count	26	Globulin level
3	Ratio of plasma albumin to globulin	11	Red blood cell distribution width -standard deviation(SD)	19	Platelet count	27	Alanine aminotransferase level
4	Absolute value of eosinophils	12	Mean hemoglobin concentration	20	Mean platelet volume	28	Absolute number of monocytes
5	Percentage of eosinophils	13	Mean hemoglobin content	21	Absolute value of basophils	29	Percentage of lymphocytes
6	Absolute value of lymphocytes	14	Uric acid level	22	Percentage of basophils	30	Hemoglobin level
7	Percentage of neutrophils	15	Albumin level	23	Microscopic red blood cell count	31	Platelet volume distribution width
8	Hematocrit	16	Absolute number of neutrophils	24	Total bilirubin level	32	Hematocrit

### Lasso Regression Algorithm

Lasso regression feature selection is an unbiased estimation used to process high-dimensional complex collinearity data. The basic idea is to construct a penalty function to select the main variables with a strong correlation with the output parameters from the input variables and build a refined regression model (18). The penalty function constructed is as follows:


$$\begin{array}{l} \hat{\beta_{0}},\hat{\beta }=\arg\min\{{\sum_{i=1}^{n}\left(y_{i}-\beta_{0}-\sum_{j=1}^{p}\beta_{j}X_{ij}\right)}^{2}\}\\ \text{ Subjectto}\sum_{\text{j}=1}^{\text{p}}\left|\beta_{\text{j}}\right|\leq \;\lambda \end{array}$$

where *y*_*i*_ is the dependent variable, *X*_*ij*_ = (*X*_*i*1_, *X*_*i*1_, …, *X*_*in*_) is an independent variable, β_*j*_ is the regression coefficient of the jth variable, and the value of λ can be [0, + ∞). Lasso feature selection compresses the model coefficients by increasing the penalty coefficient λ. When the absolute value of the regression coefficient Lasso estimate in the model is less than the absolute value of the minimum regression coefficient, some of the coefficients of the variables not strongly correlated are compressed to 0, and the variables corresponding to the coefficients with the estimated value of 0 are eliminated. In this way, the independent variables strongly related to the dependent variable are screened to achieve the purpose of feature selection. We used L1-penalized least absolute shrinkage and selection regression for multivariable analyses, augmented with tenfold cross-validation for internal validation.

### Statistical Analysis

The continuous variables were analyzed by mean ± standard deviation, and the normality was tested by the Shapiro–Wilk method. A one-way analysis of variance was used to compare the differences between the metabolome and non-metabolome biomarkers in routine physical examination. The random sampling method was used to deal with the sample imbalance between workers with and without MS (19). The area under the receiving operating characteristic curve (AUC), true positive rate (also called sensitivity or recall), and false positive rate (specificity) are represented in a graphical plot. Based on the selected biomarkers, the logistic regression model was established, and the odds ratio (OR) value of each biomarker was given. Then, we established a nomogram risk prediction model. Two criteria, the concordance index (C-index) and the calibration curve, were used to validate the prediction model in the selected biomarker sets. The C-index, a value range between 0 and 1, is to assess the performance of the model. The larger the C-index (>0.70), the better the performance of the model. Calibration curves closer to ideal ones were thought to have the accurate predictive ability of this nomogram. Furthermore, we performed decision curve analysis (DCA) to visualize the net benefit for clinical decisions.

A test *P*-value < 0.05 indicated a statistically significant difference. The Lasso algorithm used the “glmnet” package for calculation. The nomogram was developed using the packages of “rms” and “foreign.” All analyses were performed using the statistical programming environment R (version 3.6.0).

## Results

A total of 2844 occupational workers were involved (Table 2), including 655 with MS (638 men and 17 women) and 2189 without MS (1936 men and 253 women). The body weight was greater in the MS group (78.4 kg) than in the non-MS group (64.9 kg). The average systolic blood pressure was higher in the MS group (86.5/154.1 mm Hg) than in the non-MS group (72.5/118.5 mm Hg). The one-way analysis of variance revealed differences in the expression of 14 physical examination biomarkers (*P* < 0.05) (Table 3).

**Table 2:** Basic characteristics of the population.

	**Metabolic syndrome** **(*N* = 655)**	**Nonmetabolic syndrome** **(*N* = 2,189)**
**Sex**		
Male	638	1,936
Female	17	253
Age (mean, year)	26.1	25.7
Weight (mean, kg)	78.4	64.9
Height (mean, cm)	169.9	171.4
SBP/DBP (mean, mm Hg)	86.5/154.1	72.4/118.5

**Table 3:** Basic characteristics of routine physical examination markers.

	**MS** **(mean + SD)**	**Non-MS** **(mean + SD)**	***P-*value**
Red blood cell count (10^12^/*L*)	6.99 ± 1.67	5.76 ± 1.60	0.02
Total protein (g/L)	75.01 ± 3.67	73.94 ± 3.69	0.01
White blood cell ratio (10^9^/*L*)	1.67 ± 0.22	1.78 ± 0.25	0.01
Absolute value of eosinophils (10^9^/*L*)	0.17 ± 0.13	0.18 ± 0.18	0.78
Percentage of eosinophilic granule cells (%)	2.24 ± 1.36	2.38 ± 1.87	0.38
Absolute value of lymphocytes (10^9^/*L*)	2.45 ± 0.69	2.39 ± 0.71	0.37
Percentage of neutrophils (%)	57.87 ± 8.67	55.04 ± 8.51	0.01
Hematocrit	0.47 ± 0.04	0.46 ± 0.03	0.17
Large platelet ratio (%)	32.74 ± 7.33	31.79 ± 7.78	0.14
Red blood cell distribution width CV (%)	13.00 ± 1.17	12.84 ± 0.93	0.04
Red blood cell distribution width SD (fl)	41.51 ± 2.85	41.45 ± 2.78	0.77
Mean hemoglobin level (pg)	334.92 ± 11.69	336.08 ± 10.62	0.19
Mean hemoglobin concentration (g/L)	29.58 ± 2.50	29.89 ± 2.05	0.06
Uric acid level (μmol/L)	5.93 ± 0.61	5.99 ± 0.61	0.21
Albumin level (g/L)	46.74 ± 2.85	47.05 ± 2.49	0.13
Absolute number of neutrophils (10^9^/*L*)	4.32 ± 1.42	3.71 ± 1.25	0.01
Percentage of monocytes (%)	5.66 ± 1.44	5.89 ± 1.46	0.06
Leukocyte count (10^9^/*L*)	7.37 ± 1.79	6.68 ± 1.65	0.01
Platelet count (10^9^/*L*)	241.23 ± 66.96	227.95 ± 57.80	0.01
Mean platelet volume (fl)	10.97 ± 0.89	10.87 ± 0.96	0.21
Absolute value of basophils (10^9^/*L*)	0.07 ± 0.16	0.05 ± 0.11	0.02
Percentage of basophils (%)	0.28 ± 0.20	0.32 ± 0.25	0.11
Red blood cells under the microscope	2.54 ± 14.50	4.11 ± 70.52	0.78
Total bilirubin level (μmol/L)	13.81 ± 6.73	14.48 ± 6.10	0.19
Globulin level (g/L)	28.27 ± 3.02	26.89 ± 3.25	0.01
Alanine aminotransferase level (U/L)	53.48 ± 120.12	28.89 ± 29.45	0.01
Absolute number of monocytes (10^9^/*L*)	0.42 ± 0.15	0.39 ± 0.13	0.01
Percentage of lymphocytes (%)	33.90 ± 8.14	36.33 ± 7.98	0.01
Hemoglobin level (g/L)	287.23 ± 100.20	296.25 ± 92.73	0.24
Platelet volume distribution width (fl)	13.30 ± 2.01	13.16 ± 2.17	0.44
Platelet packed volume (fl)	0.27 ± 0.06	0.25 ± 0.05	0.01
Average red blood cell volume (fl)	88.22 ± 5.92	88.90 ± 5.00	0.10

**Figure 1:**
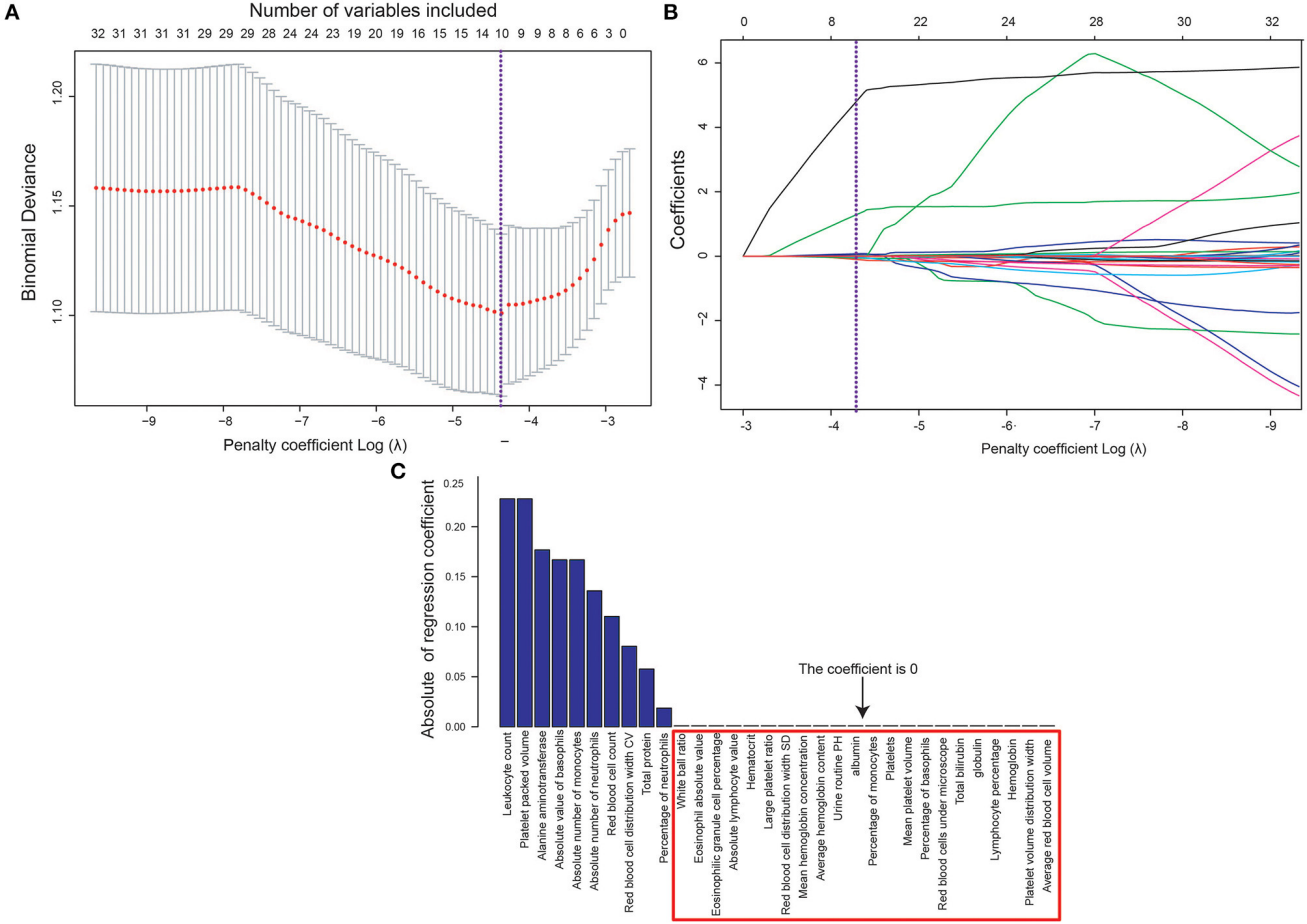
**(A)** Tuning parameter (λ) selection in the Lasso model used tenfold cross-validation based on the minimum criteria. **(B)** Changes in 32 marker coefficients with the penalty parameter (λ). **(C)** 32 marker coefficients obtained according to the selected best penalty parameter (λ).

The biomarkers were selected using the Lasso binary logistic regression model (Figure 1A). The tuning parameter (λ) selection in the Lasso model used tenfold cross-validation based on the minimum criteria. The area under the binomial deviance curve was plotted versus log (λ). Dotted vertical lines were drawn at the optimal values using the minimum criteria and the 1 standard error of the minimum criteria (the 1-SE criteria). Further, log (λ) = −4.331 was chosen (1-SE criteria) according to tenfold cross-validation of the Lasso coefficient profiles of the 32 features. A coefficient profile plot was produced against the log (λ) sequence (Figure 1B). A vertical line was drawn at the value selected using tenfold cross-validation, where optimal λ resulted in 10 nonzero coefficients. Finally, the 10 physical examination biomarkers related to MS were selected (Figure 1C). They were leukocyte count, platelet packed volume, alanine aminotransferase, absolute value of basophil, absolute number of monocytes, absolute number of neutrophils, red blood cell count, red blood cell distribution width CV, total protein, and percentage of neutrophils.

**Figure 2:**
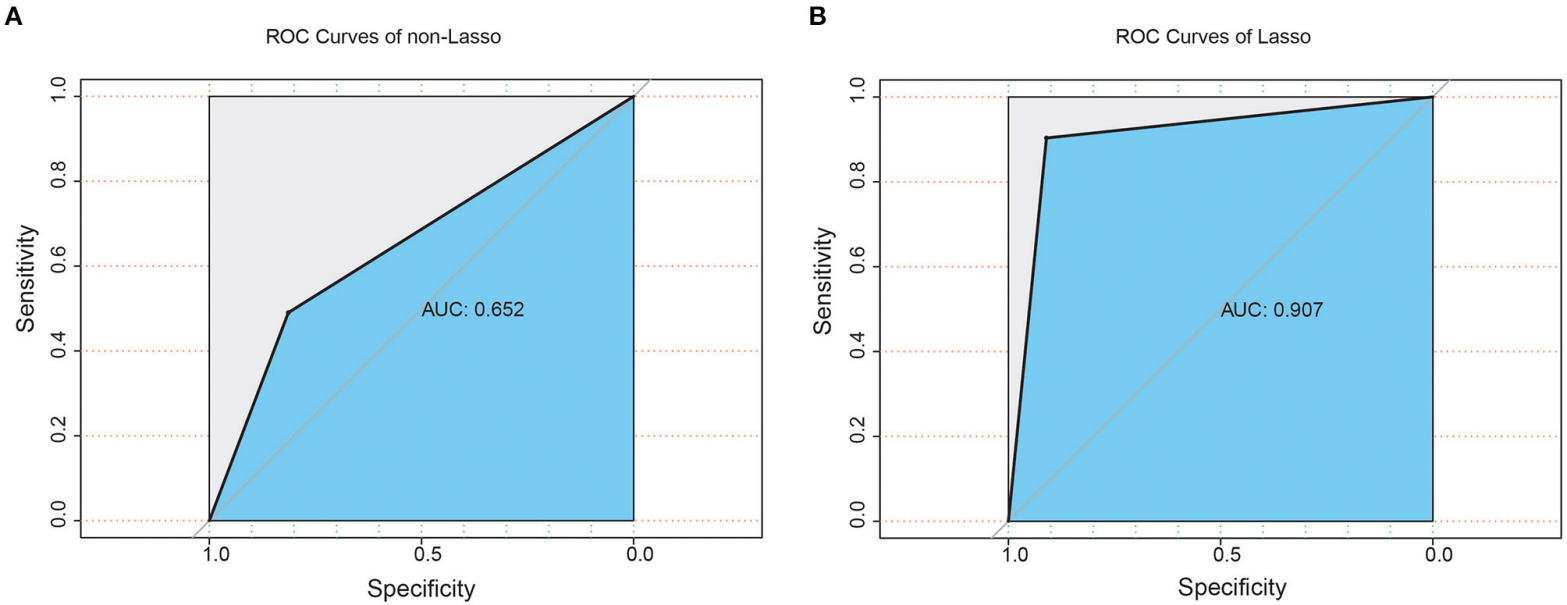
Receiver operating characteristic (ROC) curve with area under the curve values for **(A)** non-Lasso regression and **(B)** Lasso regression.

A multiple logistic regression model was established, and the accuracy of the model was compared. All 32 physical examination biomarkers were incorporated into the model. The predicted results of the model are shown in Figure 2A, indicating that the AUC of the model was 0.652 (95%CI:0.578–0.712). The prediction result of the model after incorporating the final 10 biomarkers into the model is shown in Figure 2B. The AUC of the model was 0.907 (95%CI:0.841–0.932).

**Figure 3:**
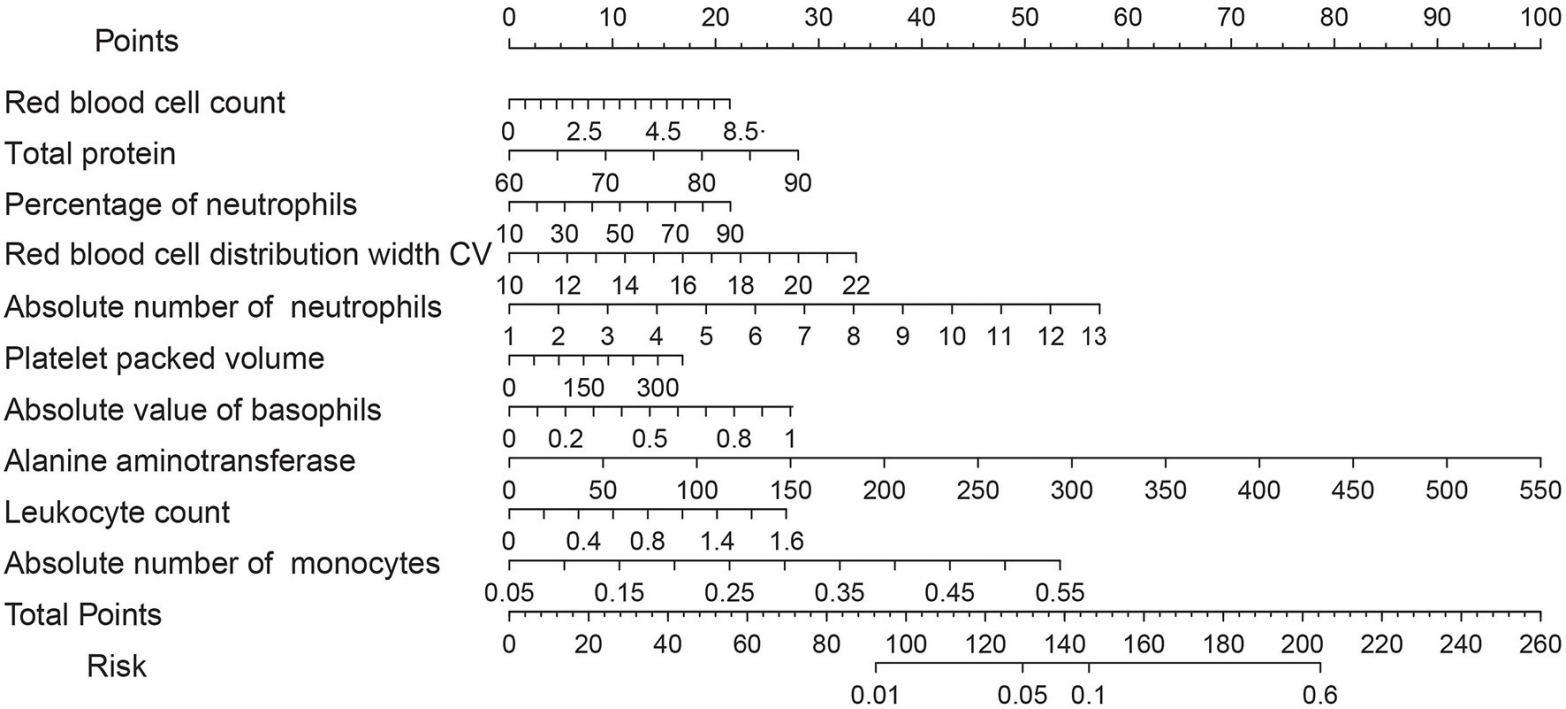
Study population for multivariate logistic regression analyses.

A multiple logistic regression model was established using the 10 physical examination biomarkers selected; the analysis results are shown in Figure 3. The following five risk factors were not associated with MS (*P* < 0.05): absolute basophil count (OR: 3.38, CI:1.05–1.98), platelet packed count (OR: 2.63, CI:2.31–3.79), leukocyte count (OR: 2.01, CI:1.79–2.19), red blood cell count(OR: 1.99, CI:1.80–2.71), and alanine aminotransferase level (OR: 1.53, CI:1.12–1.98). Only two physical examination biomarkers showed no statistical significance in the prediction model (*P* > 0.05).

**Figure 4:**
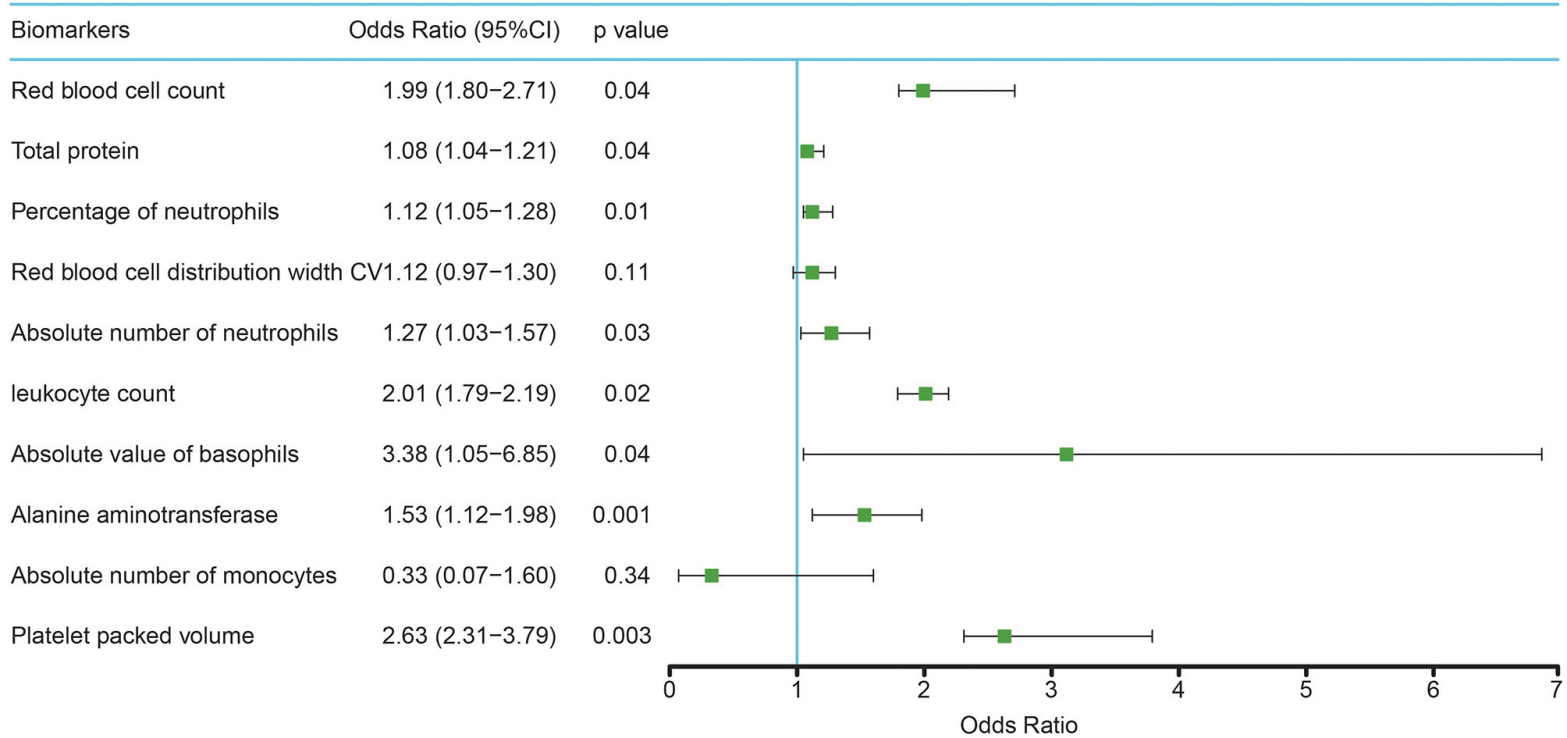
Nomogram for the prediction of MS with Lasso selected biomarkers.

**Figure 5:**
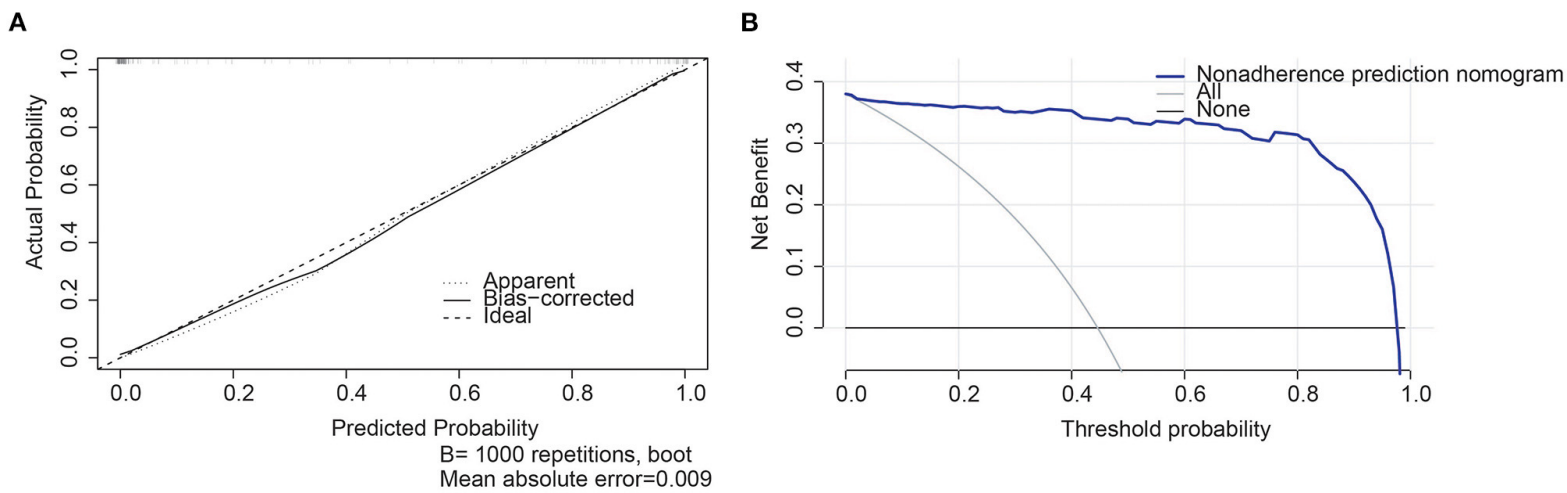
**(A)** Calibration plots for predicting MS. X-axis: bootstrap-predicted; y-axis: actual outcome, **(B)** Decision curve analysis (DCA) of the novel nomogram for predicting MS. X-axis: cut-off probability; y-axis: net benefit.

According to the selected biomarkers, we established a nomogram risk prediction model containing independent risk factors. The scores of the items displayed in the nomogram should be added up. As it is shown in Figure 4, alanine aminotransferase was associated with the highest risk, followed by the absolute number of neutrophils and the absolute number of monocytes.C-indexes were observed in both the selected biomarker sets (0.840); high agreements between ideal curves and calibration curves were observed. These results revealed a good discrimination ability of the nomogram prediction model (Figure 5A). The DCA curve revealed a more extensive range of cutoff probabilities shown by the nomogram. The threshold probabilities of the model had excellent net benefits and enhanced performance for predicting the patients with MS (Figure 5B).

## Discussion

This study selects the occupational population as the research object, with a large sample size and comprehensive inclusion indicators. We screened out 10 biomarkers related to MS in the occupational population. The established MS prediction model can be extended to clinical and physical examination centers to provide a judgment basis for the early risk assessment of MS in the occupational population.

The health of the occupational population has a strong relationship with the working environment. This population has high work pressure, disordered work and rest, irregular diet, and lack of exercise. These inevitable adverse factors increase the risk of MS (20). Hsiao and Yang conducted a 2-year (2003–2005) and 5-year (1997–2006) follow-up on a Chinese population (21). They both confirmed the routine examination of biomarkers such as serum cholesterol, triglyceride, and blood glucose levels, height, weight, blood pressure, and so forth. In this study, 10 biomarkers related to MS were further screened, including red blood cell count, total protein level, percentage of neutrophils, red blood cell distribution width CV, absolute number of neutrophils, leukocyte count, absolute value of basophils, alanine aminotransferase level, monocyte count, and platelet count. These potential biomarkers could be used to assess the risk of MS.

A low-level inflammatory state is considered to be a major potential mechanism of MS. Leukocyte is one of the most sensitive indicators reflecting inflammatory activity *in vivo*. Many studies have found that routine blood parameters are related to MS. A longitudinal cohort study of a healthy population in China showed a significant correlation between white blood cell count and MS (relative risk = 2.66). At the same time, the total numbers of white blood cells, neutrophils, monocytes, and basophils were the risk factors for obesity (22). (23) found a significant positive correlation between alanine aminotransferase level and risk of MS through quantitative and qualitative analyses, which had a predictive value for the incidence of MS (23). Further, a positive correlation was reported between red blood cell parameters, hematocrit, and MS for a large longitudinal cohort in China (24). Laufer et al. found that the prevalence of MS was 29% when the red blood cell distribution width was <14%, and the prevalence of MS was 34% when the red blood cell distribution width was more than 14% (25). Macrophage activation plays a crucial role in metabolic dysfunction, and neutrophils, as the representative of macrophages, must be closely related to metabolic syndrome (26). The findings on the biomarkers screened in the aforementioned studies were the same as those in the present study.

The research method in this paper is novel, and similar studies are rarely reported. This method effectively avoids the collinearity between independent variables so as to better screen biomarkers related to metabolic syndrome. Lasso is a method used to find out the essential structure of multivariate observation variables. However, the follow-up time of the longitudinal monitoring physical examination cohort constructed in this study is relatively short, and follow-up studies are needed to further verify the accuracy and effectiveness of the risk assessment model. In future research, we can continue to expand the sample size, verify the accuracy of the screened biomarkers, and finally establish the prediction model. We can use different research methods, such as decision trees (27), random forests (28), neural networks (29), and so forth, to compare the accuracy of each method in future studies.

## Conclusions

This study selected 10 physical examination indicators related to MS based on the Lasso algorithm. An accurate risk prediction model for MS was established. The use of common indicators and examination items in the health examination of ordinary occupational populations provides a basis for using cost-effective and portable methods to realize the risk prediction of MS.

## Data Availability Statement

The original contributions presented in the study are included in the article/supplementary material, further inquiries can be directed to the corresponding authors.

## Author Contributions

Y-RC and Z-HF conceived the study and designed the analysis. Z-YH, Y-MC, and C-JC curated the clinical data. M-WW, CW, and J-YK performed statistical analysis. Q-YX and M-WW wrote the first draft of the manuscript. X-YF and X-WZ participate in revision the manuscript. All authors contributed to revision of the manuscript.

## Funding

The presented study was supported by the Hangzhou Science and technology development plan projects (Nos. 20140633B32, 20200834M29); Youth fund of Zhejiang Academy of Medical Sciences (No. 2019Y009); Medical and Technology Project of Zhejiang Province (Nos. 2021HY127, 2020362651, and 2021KY890); Hangzhou science and Technology Bureau fund (Nos. 20191203B96, 20191203B105); Clinical Research Fund of Zhejiang Medical Association (No. 2020ZYC-A13); and Hangzhou Health and Family Planning Technology Plan key projects (No. 2017ZD02).

## Conflict of Interest

The authors declare that the research was conducted in the absence of any commercial or financial relationships that could be construed as a potential conflict of interest.

## Publisher's Note

All claims expressed in this article are solely those of the authors and do not necessarily represent those of their affiliated organizations, or those of the publisher, the editors and the reviewers. Any product that may be evaluated in this article, or claim that may be made by its manufacturer, is not guaranteed or endorsed by the publisher.

## Abbreviations

MS: Metabolic syndrome
OR: odds ratio
AUC: area under the curve
ROC: receiver operating characteristic
DCA: decision curve analysis
C-index: Concordance index.
